# Transjugular approach percutaneous closure: a preferred solution for challenging surgical management of ventricular septal rupture

**DOI:** 10.1186/s43044-025-00638-y

**Published:** 2025-04-30

**Authors:** Hendri Susilo, Emil Prabowo, Roy Bagus Kurniawan, Dian Paramita Kartikasari, Aditha Satria Maulana, Yudi Her Oktaviono

**Affiliations:** 1 Department of Cardiology and Vascular Medicine, Faculty of Medicine, Universitas Airlangga, Surabaya, Indonesia; 2 Department of Cardiology and Vascular Medicine, Dr. Soetomo General Academic Hospital, Surabaya, Indonesia; 3 Faculty of Medicine, Universitas Airlangga, Surabaya, Indonesia; 4 Department of Cardiology and Vascular Medicine, Faculty of Medicine, Universitas Jember, Dr. Soebandi General Hospital, Jember, Indonesia

**Keywords:** Transjugular approach, Percutaneous closure, Ventricular septal rupture, Failed thrombolytic, Rescue PCI

## Abstract

**Background:**

Ventricular septal rupture (VSR) is a rare but life-threatening complication following myocardial infarction (MI). While traditional management typically involves surgical repair, percutaneous closure techniques are increasingly being considered, particularly in cases where surgery is challenging or patients are high risk.

**Case presentation:**

We present the case of a 62-year-old male with anterior ST-segment elevation MI, complicated by a large VSR. Transthoracic echocardiography (TTE) revealed an 11.8-mm VSR, hypokinetic anteroseptal and anterior walls, and an ejection fraction of 52%. Surgical repair was considered high risk due to the patient’s advanced age, hypertension, anterior MI and apical VSR. Consequently, the heart team opted for a transjugular percutaneous closure approach. A 20-mm ASD occluder device was successfully deployed across the defect, as confirmed by cineangiography. Post-procedure, the patient showed clinical improvement, with resolution of the murmur and stabilization of hemodynamics. Follow-up TTE demonstrated proper occluder placement with minimal residual shunt.

**Conclusions:**

This case highlights the feasibility and effectiveness of transjugular percutaneous closure for managing complex VSR post-MI, especially in patients unsuitable for surgical repair.

**Supplementary Information:**

The online version contains supplementary material available at 10.1186/s43044-025-00638-y.

## Background

Ventricular septal rupture (VSR) is an uncommon yet critical complication of acute myocardial infarction (MI), typically manifesting within the first week post-infarction [1]. This condition arises when ischemic damage to the myocardium leads to a defect in the ventricular septum, resulting in the formation of a left-to-right intracardiac shunt. Such shunting significantly disrupts hemodynamics, often leading to rapid clinical deterioration and increased mortality rates among MI patients. Despite advancements in reperfusion strategies, such as primary percutaneous coronary intervention (PCI) and thrombolytic therapy, VSR remains a significant contributor to mortality in MI patients [2]. The formation of a new left-to-right intracardiac shunt severely compromises hemodynamics and leads to rapid deterioration [3]. Traditional management has relied heavily on surgical repair; however, the inherent risks associated with open-heart surgery, particularly in patients with multiple comorbidities, necessitate the exploration of alternative treatment strategies. In this context, custom-made occluders for VSR are available with several innovative features that offer a promising alternative to surgery [4]. Recent studies have highlighted the feasibility of percutaneous approaches, demonstrating promising outcomes in terms of mortality reduction and complication rates. These findings underscore the potential for transcatheter closure to be integrated into standard treatment protocols for VSR, especially for patients deemed high risk for surgical intervention. Long-term outcomes of percutaneous closure have shown promising reductions in mortality and complications, further supporting its adoption [5].

## Case presentation

A 62-year-old male with a history of hypertension and ischemic stroke 4 years prior was referred to our emergency department with anterior ST-elevation myocardial infarction (STEMI). The onset of his myocardial infarction occurred 36 days earlier and was complicated by ventricular septal rupture (VSR). Rescue percutaneous coronary intervention (PCI) had been performed at the referring hospital, resulting in the implantation of a drug-eluting stent (DES) measuring 2.75 × 38 mm in the left anterior descending artery (LAD) due to the failure of thrombolytics in the acute setting (Video 1). This successful reperfusion in the LAD mitigated the need for coronary artery bypass grafting (CABG), as no additional significant coronary lesions were identified.

**Fig. 1:**
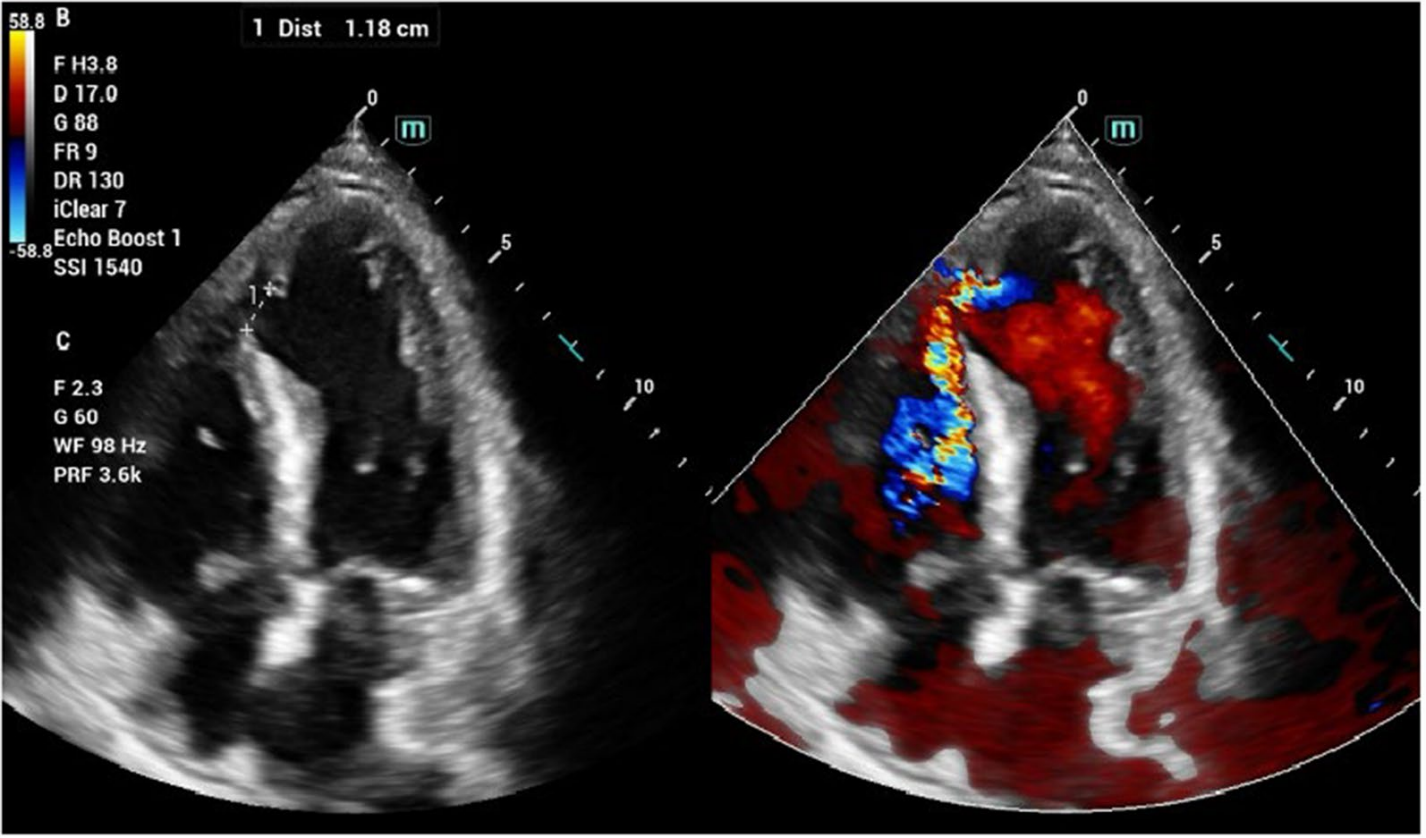
Bedside transthoracic color Doppler echocardiogram (TTE): Apical 4-chambers view shows an 11.8-mm ventricular septal rupture (VSR) located at the apical segment

However, the patient subsequently developed progressively worsening shortness of breath, accompanied by a newly noted harsh grade IV/VI holosystolic murmur at the apex. Bedside transthoracic echocardiography (TTE) demonstrated hypokinesia of the anteroseptal and anterior walls, with a preserved left ventricular (LV) ejection fraction of 52%. An echocardiogram identified a VSR with a maximal diameter of 11.8 mm (Fig. 1). Given the complex apical location of the VSR and the patient’s stable condition, the heart team opted for a transcatheter closure approach. This method, utilizing fluoroscopy and real-time transesophageal echocardiography (TEE) guidance (Video 2), was favored due to the challenges presented by the apical location for surgical closure, such as a limited surgical field of view.

**Fig. 2:**
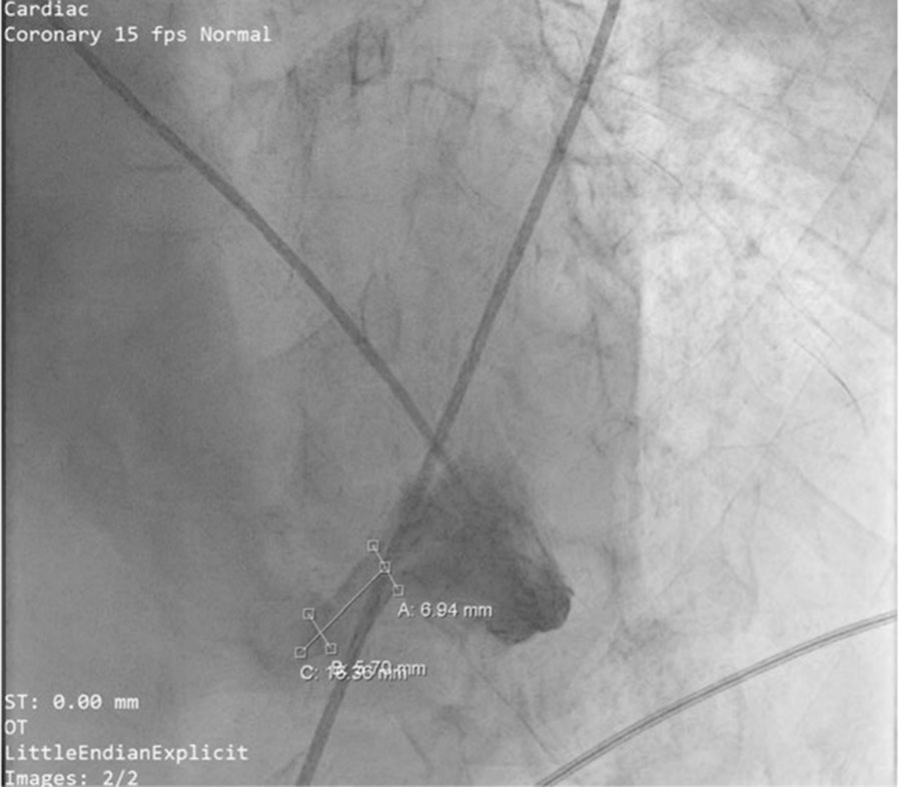
A VSR was identified via LV ventriculography in the apical area, measuring 6.94 mm × 5.70 mm × 16.36 mm

For access, a combination of the right internal jugular vein and left femoral artery was chosen, as this approach facilitated easier navigation through the right atrium, right ventricle (RV), and into the LV. A 6-F pigtail catheter was advanced via the left femoral artery into the LV for left ventriculography, and left ventricular end-diastolic pressure (LVEDP) measurements were recorded prior to the injection of 50 cc of contrast (Video 3). The ventriculography confirmed an apical VSR measuring 6.94 mm × 5.70 mm × 16.36 mm (Fig. 2).

The 7-F delivery system was advanced through the jugular approach using a 0.64 mm diameter × 180 cm Shineyard metal wire to navigate into the RV and then through the VSR into the LV cavity (Video 4). The initial attempt to place a 14 mm × 12 mm VSD occluder (Konar-FM) failed, as the device was too small for the defect (Video 5). Consequently, the team opted to use a 20-mm ASD occluder, which was readily available and adequately covered the defect (Video 6). Cineangiography confirmed proper placement of the device with minimal residual shunt (Video 7).

**Fig. 3:**
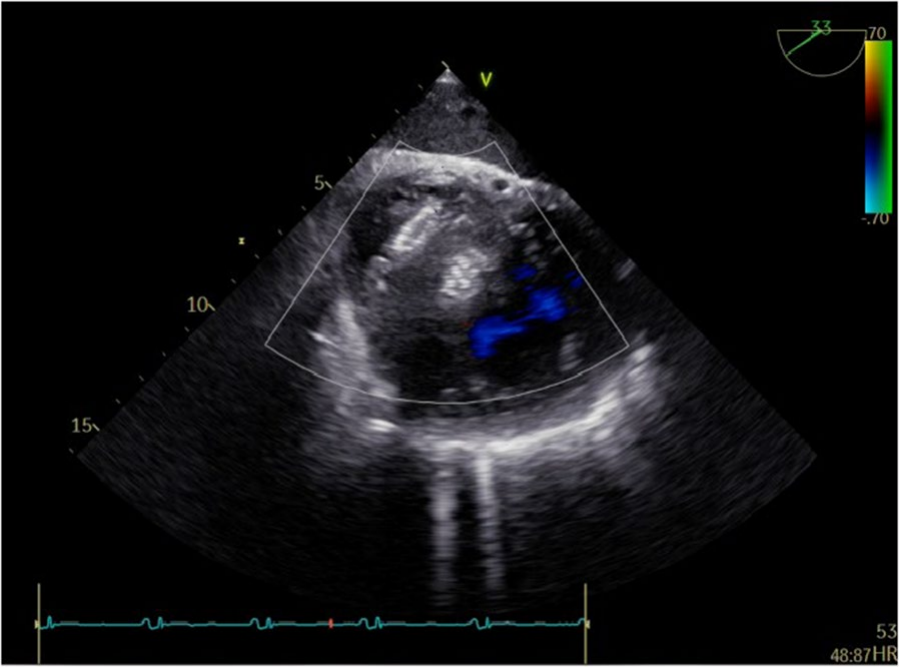
Transoesophageal echocardiography (TEE) post-procedural showed a 20-mm ASD occluder device (Ref FQFDQ-I 20, SN 24031223, Lot 202,403) was successfully placed into VSR area, with minimal residual flow

**Fig. 4:**
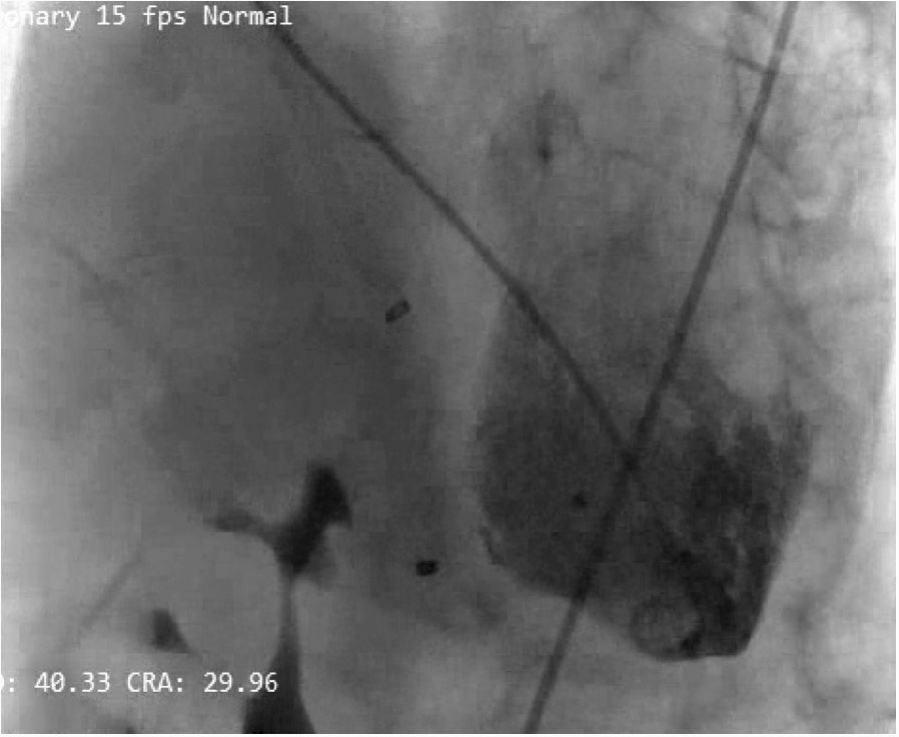
Left ventricular ventriculography showed a 20-mm ASD occluder device (Ref FQFDQ-I 20, SN 24031223, Lot 202,403) was successfully placed into the VSR area, with minimal residual flow

The transjugular approach was further validated, as it provided a technically feasible option for accessing the apical VSR. After successfully advancing the delivery system from the venous access into the LV, the deployment of the ASD occluder device was executed with precision. Cineangiography and TEE (Fig. 3) confirmed optimal device placement, with LV ventriculography (Fig. 4) showing minimal, nonsignificant residual shunt, indicating appropriate device sizing.

The patient exhibited significant hemodynamic improvement and was discharged 2 days after the procedure. Post-closure, the patient remained stable and showed no further clinical signs of heart failure, with a follow-up appointment scheduled for 1 month later. Ongoing follow-up is crucial for managing patients after percutaneous closure of ventricular septal rupture (VSR), particularly to monitor for any residual shunting. In this case, the patient is set for regular echocardiographic assessments, with the expectation that thrombus formation around the occluder will help reduce any residual flow.

**Fig. 5:**
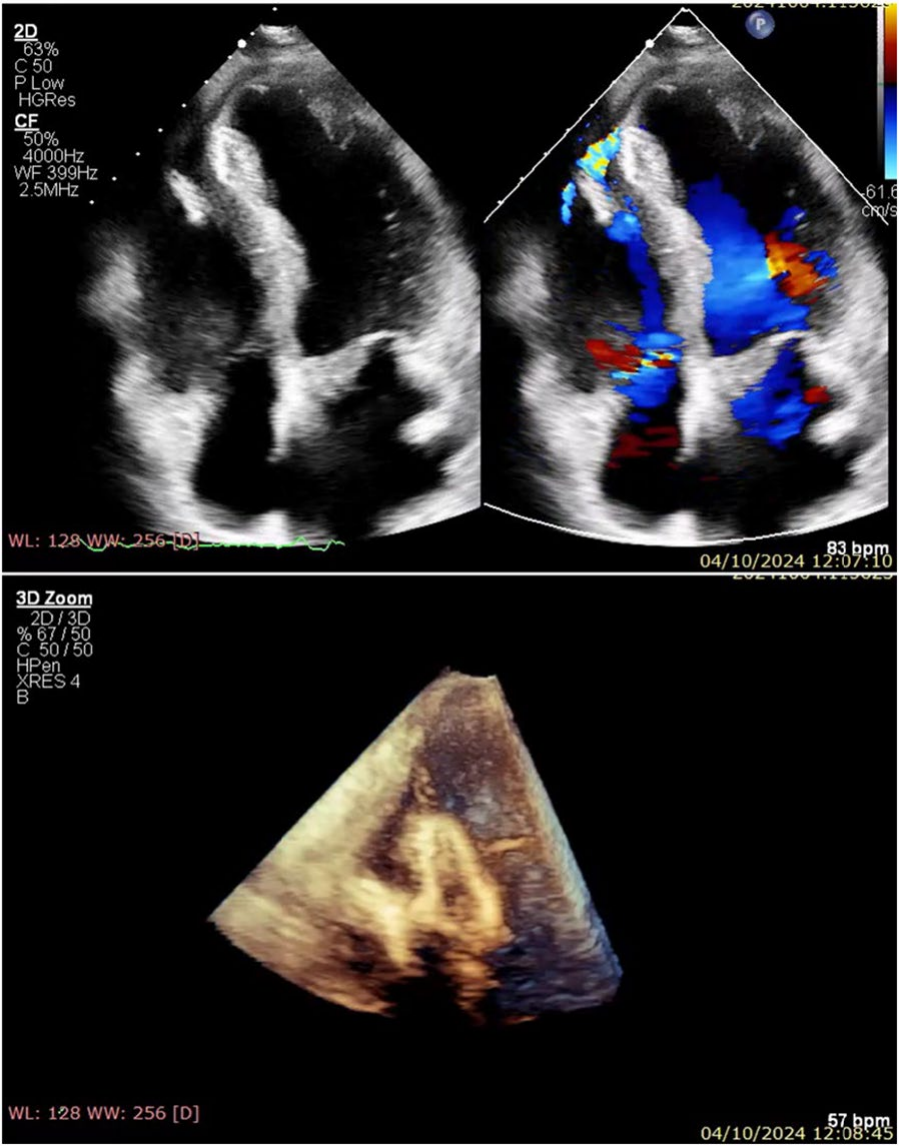
One-month follow-up echocardiographic evaluations confirmed that the occluder was properly positioned, with minimal residual flow

At the one-month follow-up, the patient’s clinical condition was stable. Echocardiographic evaluations confirmed that the occluder was properly positioned, with only minimal residual flow detected, reflecting a significant improvement compared to the immediate post-procedural findings (Fig. 5). These results support the efficacy of the transcatheter approach and highlight the importance of ongoing monitoring in this patient population.

## Discussion

VSR is an infrequent but catastrophic complication of acute myocardial infarction, particularly following anterior STEMI [6, 7]. While its incidence has significantly decreased due to widespread adoption of revascularization strategies (from ∼1–2 to ∼0.25%), the risk of VSR remains in a subset of patients, especially those with delayed reperfusion or failed thrombolysis [6]. In the case we present, although rescue PCI was successfully performed with a DES in the LAD, the subsequent rupture of the interventricular septum complicated the clinical course (Fig. 1).

Our patient, who had a history of anterior STEMI, underwent rescue PCI after failed thrombolysis. Interestingly, in this case, the VSR manifested with acute heart failure and a pansystolic murmur at the apex four weeks after previous hospital discharge. VSR was confirmed at a secondary hospital, where the patient experienced worsening dyspnea and was referred to our tertiary hospital on day 36 with initial unstable hemodynamic status. Once a suspected VSR was confirmed by TTE, defect closure was advised [7, 8].

Surgical repair has long been the preferred treatment for VSR, as recommended by the guidelines of both the European Society of Cardiology and the American College of Cardiology [7–9]. However, this recommendation has not been supported by robust evidence due to the rarity of VSR in clinical practice and the limited data available from single-center experiences [10–13]. Therefore, the decision on closure strategies should tailored to the clinical condition.

Despite timely surgical intervention, a recent meta-analysis has reported in-hospital mortality rates for VSR of approximately 40%, with even higher rates in patients presenting with cardiogenic shock [12]. While immediate closure of VSR is advised, procedures performed within the first 24 h carry a mortality rate of 60% [10]. Surgeons are increasingly adopting a delayed surgical approach, particularly for hemodynamically stable patients. Mortality rates improve with delayed surgery—47% within the first week, 30% by the third week, and 10% for elective procedures performed after 21 days [10, 14]. Moreover, this surgical approach can be particularly challenging when the rupture occurs in the apical region, as in our case (Figs. 1 and 2). Apical VSR presents significant technical difficulties, including poor surgical visibility and limited access to the defect. Meanwhile, the patient was also maintained in a stable hemodynamic condition, even without pharmacological or mechanical circulatory support.

In recent years, percutaneous VSR closure has emerged as a promising alternative, particularly for patients at high risk of surgical closure, either as definitive therapy, bridge to surgery after stabilization, or correction of re-shunting due to patch leak [5]. For non-shock patients undergoing primary percutaneous closure around a median of 6.5 days post-VSR, mortality rates decrease to 38%, which is comparable to the surgical mortality observed in patients undergoing repair between 8 and 21 days (30%) [10, 15]. The current patient’s condition also favors percutaneous closure, as the VSR was identified 36 days after the onset of STEMI, which was subsequently adequately managed and stabilized. Literature, indeed, suggests that the occurrence of VSR typically peaks in the first 24 h or during the first week post-onset [8, 12]. Therefore, at the time of admission to our tertiary hospital (day 36), it was approximately 3–4 weeks since the presumed VSR occurrence (subacute VSR). With this timepoint, it was assumed that the infarct margins have evolved and stabilized, making device placement more optimal with a lower risk of defect enlargement, which could result in persisted post-procedural shunting [6, 16]. In line with our consideration, a series from China supported that percutaneous closure has a higher technical success rate when performed in a subacute setting compared to an acute one (97% vs. 78%). Moreover, the defect size observed from both echocardiography and ventriculography was less than 15 mm, which is considered in optimal range for percutaneous closure according to current recommendations [6, 17]. Diagnostic coronary angiography also revealed complete revascularization from the rescue PCI, showing only single vessel disease (in LAD). Thus, CABG was deemed unnecessary, and surgery was considered too invasive. The planned closure strategy, indeed, relies on several considerations, such as the complexity of the defect, the availability of appropriate device sizes, the speed of the procedure, the experience of the interventionist, the patient’s hemodynamic status, and the time from diagnosis to VSR occurrence [5]. This made the heart team’s decision to opt for percutaneous closure both logical and evidence-based.

The choice of vascular access is crucial in percutaneous VSR closure, with either the femoral or internal jugular vein being used for device delivery [8]. While the femoral vein is more commonly employed, the transjugular approach was considered more appropriate in this case. Drawing from experience with congenital muscular VSD, the internal jugular vein may be advantageous for treating muscular VSD closer to the apex [18]. Our interventionists considered that the jugular vein provided better angulation, enabling smoother catheter navigation from the right atrium to the right ventricle and through the VSR into the left ventricle. Thus, vascular access should be tailored to the specific clinical scenario.

Another challenge in this case was the selection of the closure device. Initially, we deployed muscular VSD occluder device. Nevertheless, the attempt was unsuccessful as too small to ensure adequate closure with subsequent device pull out. As a result, an ASD occluder, the largest device available in our center, was used to provide better closure (Figs. 3 and 4). Although not ideal, it effectively reduced the shunt with acceptable residual flow.

## Conclusions

In summary, VSR remains a serious complication, even after successful rescue revascularization and hospital discharge. If not promptly identified, VSR can significantly worsen a patient’s prognosis. Once diagnosed, immediate closure should be planned to optimize outcomes. While surgery remains the gold standard, percutaneous closure has emerged as a valuable alternative. It serves as either a primary treatment or a bridge, especially for non-surgical candidates with stable conditions. As additional insight, apical VSR presents unique surgical challenges due to suboptimal visibility and difficult access, making percutaneous closure a less invasive and feasible option, complemented by the transjugular approach for device delivery. Device selection must be adapted to what is available, with the goal of reducing shunt flow to improve clinical outcomes. Regular echocardiographic follow-up is recommended to monitor shunt reduction, with the expectation that thrombus formation around the occluder will further reduce residual shunting over time.

## Supplementary Information

Supplementary material 1.

Supplementary material 2.

Supplementary material 3.

Supplementary material 4.

Supplementary material 5.

Supplementary material 6.

Supplementary material 7.

## Data Availability

No datasets were generated or analyzed during the current study.

## Abbreviations

VSR: Ventricular septal rupture
ASD: Atrial septal defect
PCI: Percutaneous coronary intervention
CABG: Coronary artery bypass grafting
DES: Drug-eluting stent
